# Genome-Wide Identification and Expression Analysis of the *HDAC* Gene Family in *Stevia rebaudiana*

**DOI:** 10.3390/ijms27209177

**Published:** 2026-10-16

**Authors:** Chaoyong Zhao, Yaning Fu, Daiqi Wang, Kai Lin, Lingling Yun, Fen Xie, Genxiang Bai

**Affiliations:** 1 Beijing Life Science Academy, Beijing 102206, China; chaoyongzhao@stu.scau.edu.cn (C.Z.); fuyn@blsa.com.cn (Y.F.); yunll@blsa.com.cn (L.Y.); xief@blsa.com.cn (F.X.); 2 Hubei Shizhen Laboratory, School of Basic Medical Sciences, Hubei University of Chinese Medicine, Wuhan 430065, China; 3 College of Agriculture, South China Agricultural University, Guangzhou 510642, China; 4 South China Botanical Garden, Chinese Academy of Sciences, Guangzhou 510650, China; wangdaiqi@scbg.ac.cn (D.W.); linkai@scbg.ac.cn (K.L.)

**Keywords:** *Stevia rebaudiana*, histone deacetylase, epigenetic regulation, stress tolerance

## Abstract

Histone deacetylases (HDACs) are crucial epigenetic regulators that modulate chromatin structure and gene expression through histone deacetylation. *Stevia rebaudiana*, a natural source of high-intensity sweeteners, faces significant cultivation challenges due to environmental stresses such as osmotic stress and salinity. However, the HDAC gene family in *S. rebaudiana* remains largely uncharacterized. Here, we present the first genome-wide analysis of the HDAC family in *S. rebaudiana*, identifying 22 SrHDAC transcripts derived from 18 gene loci, which were classified into three subfamilies (RPD3/HDA1, SIR2, and HD2). Systematic characterization of gene structure, conserved domains, and promoter cis elements indicated their potential involvement in hormone signaling and abiotic stress regulation. Expression profiling revealed tissue-specific expression patterns and differential responses to methyl jasmonate (MeJA) treatment and PEG-induced osmotic stress. Subcellular localization of representative proteins in *Nicotiana benthamiana* leaf cells supported nucleolar-associated functions. These findings provide a foundation for understanding *HDAC*-mediated epigenetic regulation in *S. rebaudiana* growth, development, and stress adaptation, and identify candidate genes for future functional studies toward stress-tolerant cultivar improvement.

## 1. Introduction

Histone acetylation is a reversible epigenetic modification that dynamically regulates chromatin structure and gene expression. Histone deacetylases (*HDACs*) catalyze the removal of acetyl groups from lysine residues on histone tails, thereby increasing histone DNA affinity, compacting chromatin, and generally repressing transcription [1]. In plants, HDACs are classified into three major families: the RPD3/HDA1 family, the SIR2 family, and the plant-specific HD2 family [2,3,4]. Through modulation of histone acetylation status, these enzymes influence diverse biological processes, including plant growth and development, stress responses, and secondary metabolite biosynthesis [5]. Genome-wide analyses have identified variable HDAC repertoires across plant species, ranging from 14 members in Tartary buckwheat (*Fagopyrum tataricum*) to 26 in apple (*Malus pumila*) [6,7], with 19 members characterized in sorghum (*Sorghum bicolor*) [8], suggesting lineage-specific expansion and functional diversification [9].

Accumulating evidence indicates that HDACs serve as critical integrators of development and environmental signals. The RPD3/HDA1 clade regulates plant growth and stress responses, as evidenced by the developmental and physiological defects of *Arabidopsis HDA6* and *HDA19* loss-of-function mutants [10,11]. HD2 family members modulate plant growth, development, and abiotic stress tolerance, as demonstrated by *AtHD2D*-overexpressing *Arabidopsis* lines [12]. HDACs also modulate secondary metabolite accumulation: in apple, *MdHDA6* contributes to drought and salt tolerance through chromatin remodeling [7], while in tea, *CsHDA6* mediates the biosynthesis of the anti-insect metabolite α-farnesene through interaction with the transcription factor *CsMYC2* [13]. Furthermore, HDACs participate in abiotic stress adaptation. In rice, multiple RPD3/HDA1-type HDACs, including *HDA704*, *HDA710* and *HDA706*, enhance drought or salt tolerance through chromatin remodeling and the modulation of ABA signaling and stress-responsive gene expression [14,15,16], HDAC-mediated chromatin remodeling also contributes to salinity adaptation in *Arabidopsis* and rice [10,17]. These findings establish that HDACs function as versatile regulators linking epigenetic modification to plant fitness under changing environments [18].

*S. rebaudiana*, a perennial herb native to South America, is cultivated worldwide as a natural source of steviol glycosides (SGs), high-intensity sweeteners with minimal caloric content [19]. SGs are approximately 150–300 times sweeter than sucrose yet non-glycemic, making them valuable for sugar-free food and pharmaceutical applications [20]. However, *S. rebaudiana* cultivation faces significant environmental constraints. In major production regions such as northwestern China, salinity and drought substantially limit yield and quality, necessitating the development of stress-tolerant cultivars. While HDACs have been established as key epigenetic regulators of plant stress responses in model and crop species [21,22], the HDAC family members in *S. rebaudiana* remain uncharacterized. This knowledge gap limits understanding of epigenetic stress adaptation mechanisms in this economically important species and impedes molecular breeding efforts toward improved stress tolerance.

In this study, we conducted a genome-wide identification and characterization of the HDAC gene family in *S. rebaudiana*. We identified 22 HDAC transcripts derived from 18 gene loci, classified them into three subfamilies, and analyzed their phylogenetic relationships, gene structures, conserved domains, and chromosomal distributions. Furthermore, we examined cis-acting regulatory elements in promoter regions, profiled expression patterns across tissues and under methyl jasmonate (MeJA) and PEG-induced osmotic stress treatments, and determined the subcellular localization of representative members. These findings provide a systematic foundation for elucidating HDAC functions in *S. rebaudiana* and identify candidate genes for future functional validation.

## 2. Results

### 2.1. Physicochemical Properties and Subcellular Localization of SrHDAC Proteins

Homology-based searches against *Arabidopsis* HDAC sequences followed by Pfam domain validation identified 22 HDAC protein sequences, derived from 18 gene loci in the *S. rebaudiana* genome. These genes were designated *SrHDA1-10*, *SrHDT1-4*, and *SrSRT1-4* (Table S1). The coding sequences (CDS) ranged from 480 bp (*SrHDA10*) to 1983 bp (*SrHDA6* and *SrHDA7*), encoding proteins of 160–661 amino acids with theoretical molecular masses of 18.44–73.72 kDa (Table S1). The isoelectric points (pI) spanned from 4.55 (*SrHDA9*) to 9.14 (*SrSRT3* and *SrSRT4*); most members were acidic, whereas SIR2 subfamily members were predominantly alkaline. The grand average of hydropathicity (GRAVY) values ranged from −1.253 (*SrHDT1*) to 0.099 (*SrHDA4*), with 20 of 22 proteins exhibiting negative values, indicating a predominantly hydrophilic character (Table S1). These physicochemical properties were largely conserved within subfamilies: HD2 members were uniformly acidic and hydrophilic, SIR2 members were predominantly basic, and RPD3/HDA1 members displayed the broadest range of molecular weights and pI values.

Subcellular localization prediction revealed distinct distribution patterns among the three subfamilies. RPD3/HDA1 members were distributed across chloroplast (4), nucleus (4), cytoplasm (2), and cytoskeleton (3). All HD2 members (*SrHDT1-4*) were predicted to localize exclusively to the nucleus, whereas SIR2 members were found in chloroplast (*SrSRT1*), cytoplasm (*SrSRT2*), and nucleus (*SrSRT3* and *SrSRT4*). Alternative splicing isoforms of *SrHDA2* and *SrSRT1* shared identical subcellular localization predictions with their corresponding loci, whereas the *SrHDA3* and *SrHDA10* isoforms showed differential localization (chloroplast versus cytoskeleton for *SrHDA3*; nucleus versus cytoplasm for *SrHDA10*). The predominantly nuclear prediction for HD2 and a subset of RPD3/HDA1 and SIR2 members aligns with their canonical function in chromatin regulation, whereas the chloroplast and cytoplasmic predictions for other members suggest potential non-histone substrates or moonlighting functions.

### 2.2. Phylogenetic Relationship Analysis of SrHDAC Proteins

**Figure 1:**
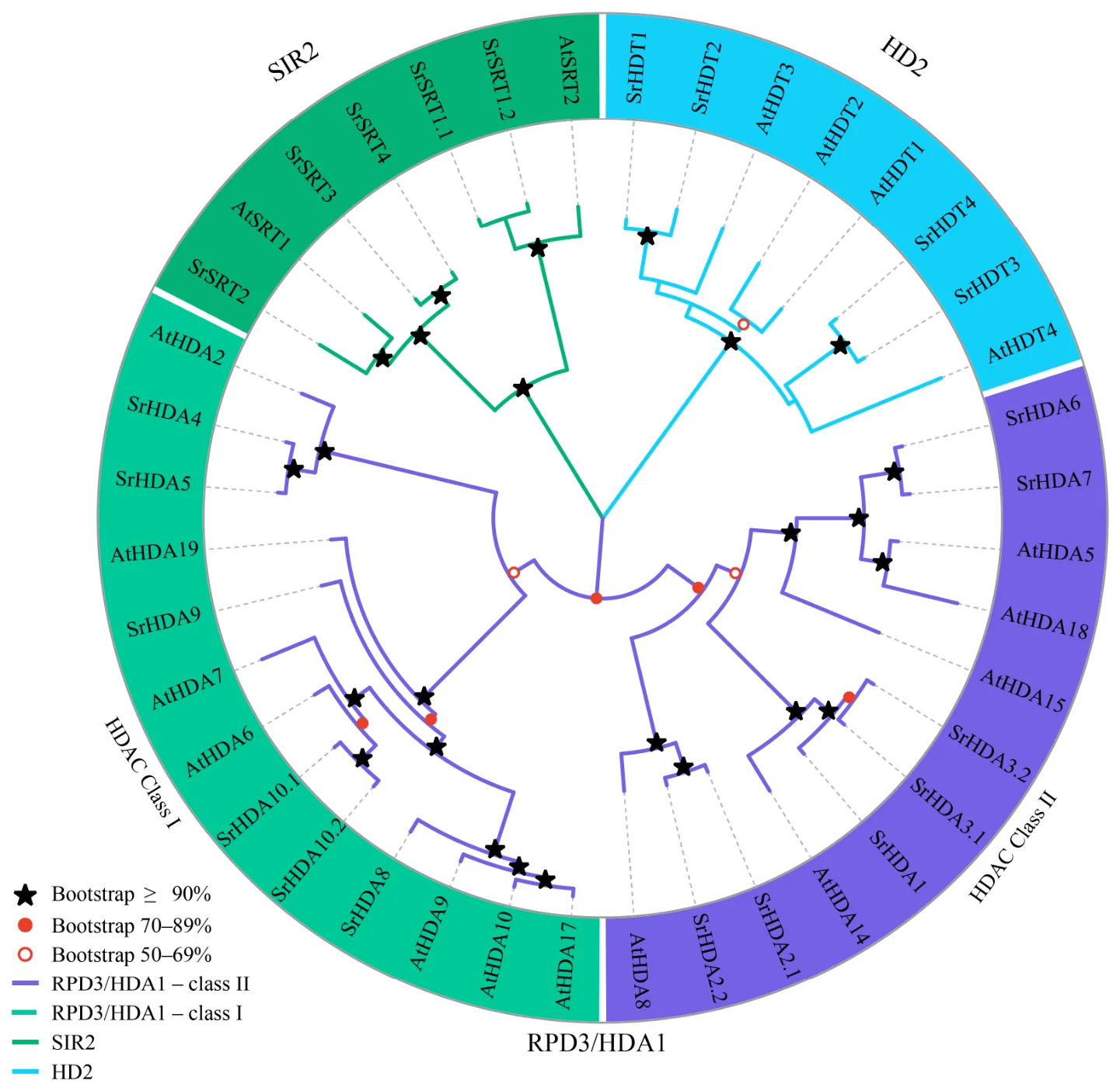
Maximum likelihood phylogeny of the HDAC superfamily in *S. rebaudiana* and *A. thaliana*. The tree was inferred from full-length protein sequence alignments (MAFFT L-INS-i) using the Maximum Likelihood method with the VT + G4 + I best-fit substitution model and 1000 bootstrap replicates; the circular phylogenetic tree resolved three well-supported clades corresponding to the RPD3/HDA1 (purple, HDAC class II; turquoise, HDAC class I), SIR2 (green) and HD2 (cyan) subfamilies, and the same colour scheme is applied to the tree branches and the outer ring background. Bootstrap support values are indicated by black stars (≥90%), red filled circles (70–89%), and red open circles (50–69%).

A maximum likelihood phylogenetic tree was constructed using full-length protein sequences of *S. rebaudiana* (22 sequences) and *A. thaliana* (18 sequences). The tree resolved three well-supported subfamilies: RPD3/HDA1 (13 members), SIR2 (5 members), and HD2 (4 members), consistent with the *Arabidopsis* classification (Figure 1). The RPD3/HDA1 subfamily was further divided into two clades, with HDAC_class II containing the largest number of members (7). The SIR2 subfamily formed a distinct monophyletic group, while the HD2 subfamily clustered separately, reflecting its plant-specific origin. These relationships suggest lineage-specific expansion of the HDAC family in *S. rebaudiana*, particularly within the RPD3/HDA1 subfamily.

### 2.3. Gene Structures and Conserved Domains of SrHDAC Members

**Figure 2:**
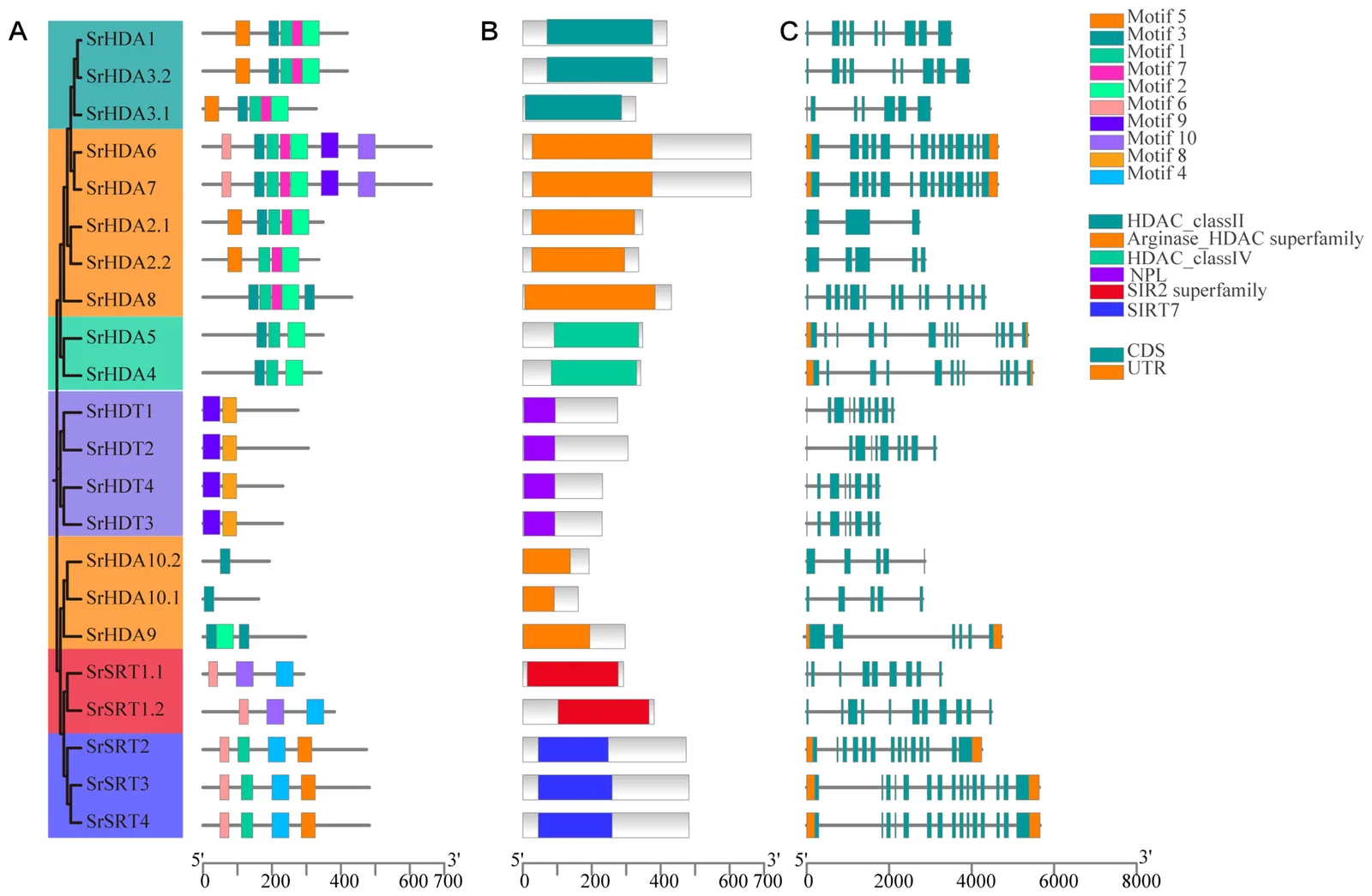
Structural characterization of the *SrHDAC* gene family. (**A**) Distribution of 10 conserved protein motifs across *SrHDAC* members, with different colored boxes representing distinct motifs (Motif 1–10) identified by MEME analysis. (**B**) Domain architecture of SrHDAC proteins, showing the characteristic domains identified by CDD annotation: HDAC_classII, Arginase_HDAC superfamily, HDAC_classIV, NPL, SIR2 superfamily, and SIRT7, color-coded as indicated in the panel legend. Gray bars indicate relative protein lengths. (**C**) Exon–intron organization of *SrHDAC* genes, with blue boxes representing coding sequences (CDS), orange boxes marking 5′- and 3′-UTR regions, and gray lines indicating introns. The scale bar indicates genomic length in base pairs.

MEME analysis identified ten conserved protein motifs, and CDD classification revealed subfamily-specific domain architectures. SrHDA proteins contained motifs 1, 2, 3, 5, 6, 7, 9 and 10, corresponding to the Hist_deacetyl domain (PF00850), SrSRT proteins possessed motifs 1, 4, 5, 6, and 10, representing the SIR2 domain (PF02146). SrHDT proteins exhibited motifs 8 and 9, characteristic of the HD2 domain (PF10243) (Figure 2A,B). In addition, CDD annotation identified HDAC_classII, HDAC_classIV, Arginase_HDAC superfamily, NPL, SIR2 superfamily, and SIRT7 domain signatures, which were used to refine the subfamily classification (Figure 2B). These distinct motif compositions across subfamilies reflect their specialized roles in histone deacetylation. Exon–intron architecture analysis revealed significant structural diversity among subfamilies. RPD3/HDA1 members contained 3–18 exons, SIR2 members 9–15 exons, and HD2 members 7–10 exons (Figure 2C). This structural conservation within subfamilies indicates that gene architecture is closely associated with phylogenetic relationships.

### 2.4. Chromosomal Distribution and Gene Duplication Analysis

**Figure 3:**
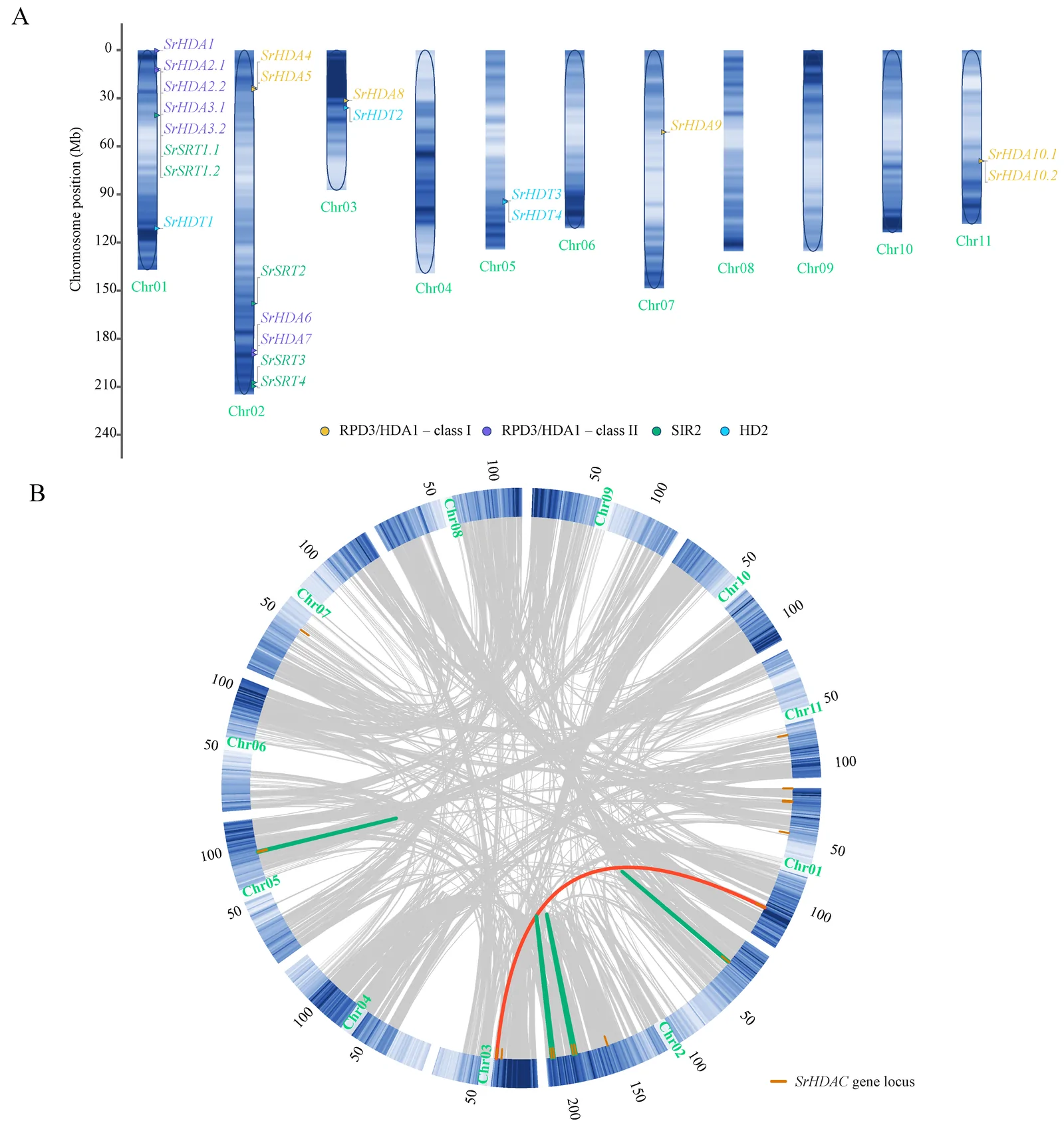
Chromosomal distribution and duplication patterns of *SrHDAC* genes in *S. rebaudiana*. (**A**) Genomic localization of 22 *SrHDAC* transcripts (18 gene loci) across 11 chromosomes. (**B**) Circos diagram illustrating interchromosomal relationships relationships and gene duplication events. Colored blocks represent individual chromosomes; gray lines indicate collinear relationships between chromosomes; green arcs denote tandem duplication pairs (*SrHDT3/SrHDT4*, *SrHDA4/SrHDA5*, *SrHDA6/SrHDA7*, and *SrSRT3/SrSRT4*), and red arcs denote the segmental duplication pair (*SrHDT1*/*SrHDT2*).

The 22 SrHDAC transcripts (18 gene loci) exhibited a non-random, uneven distribution across the *S. rebaudiana* genome. Chr01 and Chr02 harbored the highest densities, accommodating 8 and 7 genes, respectively, collectively representing 68.2% (15/22) of the family (Figure 3A). Chr03, Chr05, and Chr11 each contained 2 genes, Chr07 possessed 1. Chr04, Chr06, Chr08, Chr09 and Chr10 contained no *SrHDAC* genes.

Collinearity analysis using MCScanX identified five gene duplication pairs: four tandem duplications (*SrHDT3/SrHDT4*, *SrHDA4/SrHDA5*, *SrHDA6/SrHDA7*, and *SrSRT3/SrSRT4*), and one segmental duplication (*SrHDT1/SrHDT2*) (Figure 3B). The Ka/Ks ratios for all duplicated pairs with defined values were less than 1 (range: 0.21–0.88) (Table S2). The Ks values of the retained pairs ranged from 0.003 to 0.664; assuming a plant synonymous substitution rate of 6.1 × 10^−9^ substitutions per site per year [23], the segmental pair *SrHDT1/SrHDT2* (Ks = 0.664) was estimated to have diverged approximately 54 million years ago (Mya), whereas the four tandem pairs (Ks = 0.003–0.033) diverged within the last 0.25–2.7 Mya, indicating very recent local duplication events. The prevalence of tandem duplications on Chr02 and Chr05 likely contributed to the local expansion of the family observed on these chromosomes. Importantly, all five duplication pairs consist of two distinct gene loci rather than alternative splicing isoforms of a single locus, confirming that they represent bona fide duplication events retained in the genome [23].

### 2.5. Analysis of Cis-Acting Regulatory Elements in SrHDAC Promoters

**Figure 4:**
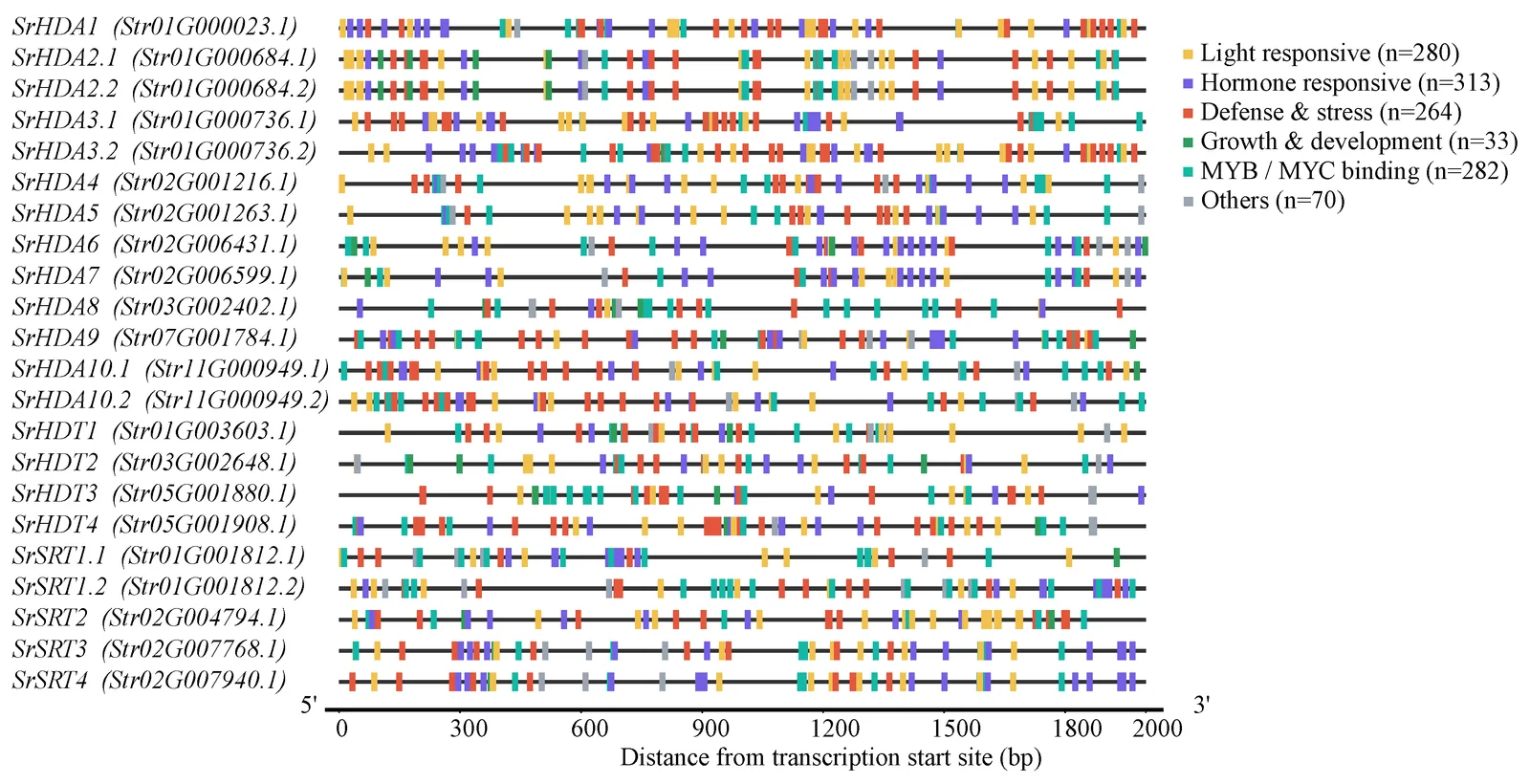
Cis-acting regulatory elements in the promoters of *SrHDAC* genes. Distribution of predicted cis-acting elements across the 2000 bp promoter regions of 22 *SrHDAC* genes. Horizontal black lines represent promoter lengths.

Analysis of 2000 bp upstream regions using PlantCARE identified diverse cis-acting elements across all 22 *SrHDAC* promoters (Figure 4; Table S3). Light-responsive elements were highly abundant category (*n* = 280), present in all promoters, with GT1-motif (*n* = 57), G-box (*n* = 53), and Box 4 (*n* = 48) being the predominant types. The ubiquitous distribution of light-responsive elements suggests that *SrHDAC* expression may be broadly regulated by light signaling.

Hormone-responsive elements were widely distributed. MeJA-responsive elements were the most abundant (*n* = 88, representing 44 unique physical sites due to overlapping CGTCA-motif and TGACG-motif annotations), followed by ABA-responsive elements (*n* = 72). Aux (*n* = 15), SA (*n* = 21), and GA (*n* = 23) responsive elements were also identified. All 22 promoters contained at least one hormone-responsive element. The promoters of *SrSRT2, SrSRT3, SrSRT4,* and *SrHDT2* contained all five hormone-responsive element types (MeJA, ABA, Aux, SA, and GA), suggesting the potential for co-regulation by multiple hormone signals, although experimental validation is required to confirm this prediction (Figure 4, Table S3).

Stress-responsive elements included anaerobic induction-responsive elements (ARE) in all 22 *SrHDAC* promoters and drought-responsive elements (MBS) in 12 promoters. Low-temperature responsive elements (LTR) were absent in *SrSRT3*, *SrSRT4* and *SrHDT2* promoters but present in the other 19 genes (Figure 4, Table S3). Growth and development-related elements, including circadian (*n* = 4), endosperm expression (*n* = 4), and meristem expression (*n* = 2) motifs, were also identified. The differential distribution of stress-responsive elements among subfamilies was observed: ARE elements were present in all RPD3/HDA1, HD2, and SIR2 promoters, whereas MBS elements were concentrated in RPD3/HDA1 promoters (8 of 13) compared to SIR2 (2 of 5) and HD2 (2 of 4).

### 2.6. Expression Analysis of SrHDAC Genes in Different Tissues

**Figure 5:**
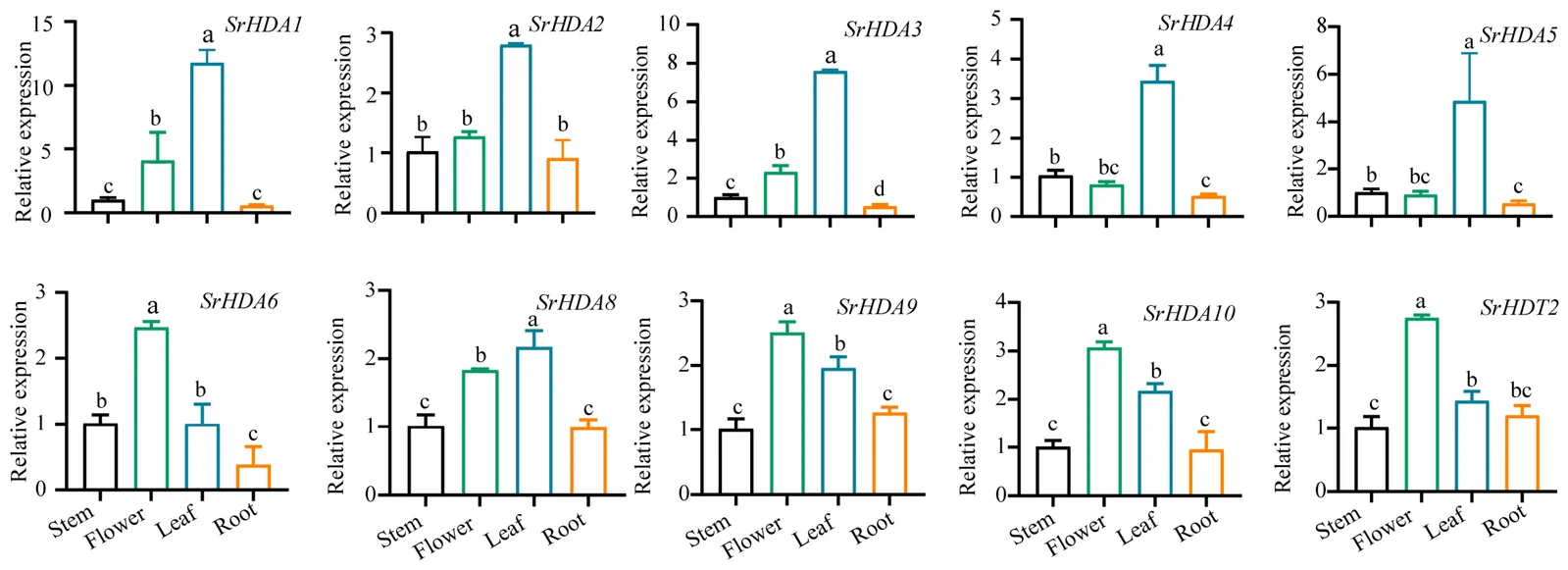
Tissue-specific expression profiles of *SrHDAC* genes in *S. rebaudiana*. Relative expression levels of 10 *SrHDAC* genes across four tissues: stem, flower, leaf, and root. Expression values were normalized to the stem (set to 1) with *SrActin* as the internal reference gene. Data represent means ± SD (*n* = 3). Statistical significance was determined by one-way ANOVA followed by LSD post-hoc test; different letters indicate significant differences at *p* < 0.05.

Quantitative RT-PCR analysis of ten representative *SrHDAC* genes across roots, stems, leaves, and flowers revealed distinct tissue-specific expression patterns (Figure 5). Six genes (*SrHDA1*, *SrHDA2*, *SrHDA3*, *SrHDA4*, *SrHDA5*, and *SrHDA8*) showed preferential expression in leaves, with transcript levels significantly higher than in other tissues. Four genes (*SrHDA6*, *SrHDA9*, *SrHDA10*, and *SrHDT2*) exhibited highest expression in flowers, suggesting potential roles in reproductive development. *SrHDA9* and *SrHDA10* were also expressed at substantial levels in leaves; a dual-expression pattern that may reflect their involvement in the developmental transition from vegetative growth to flowering or in coordinating physiological processes shared between photosynthetic and reproductive tissues.

### 2.7. The Responses of SrHDAC to MeJA Induction

**Figure 6:**
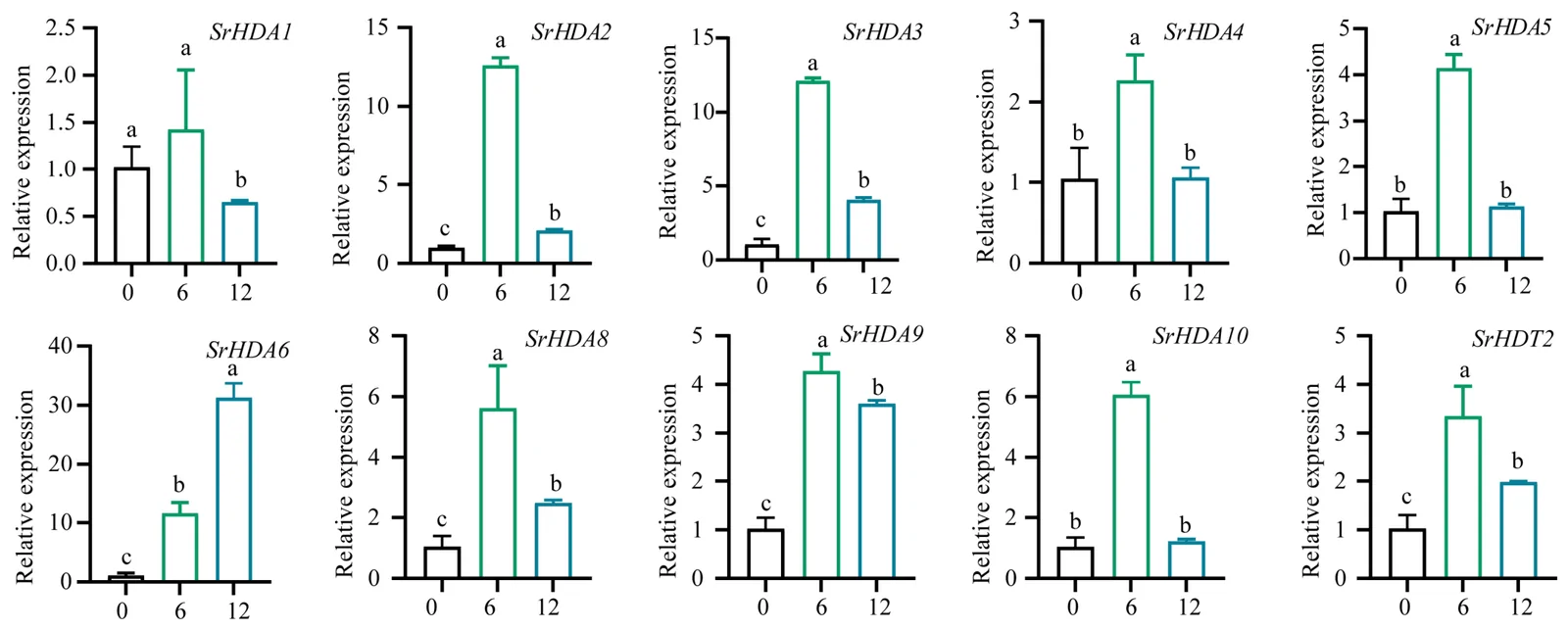
Expression dynamics of *SrHDAC* genes in response to MeJA treatment. Relative expression levels of ten *SrHDAC* genes in 60-day-old *S. rebaudiana* plants treated with methyl jasmonate (MeJA) for 0 (control), 6, and 12 h. Expression values were normalized to the 0 h control (set to 1) with *SrActin* as the internal reference gene. Data represent means ± SD (*n* = 3). Statistical significance was determined by one-way ANOVA followed by LSD post-hoc test; different letters indicate significant differences at *p* < 0.05.

Temporal expression analysis following MeJA treatment revealed differential responses among the ten examined *SrHDAC* genes (Figure 6). Nine genes (*SrHDA2*, *SrHDA3*, *SrHDA4*, *SrHDA5*, *SrHDA6*, *SrHDA8*, *SrHDA9*, *SrHDA10*, and *SrHDT2*) were significantly upregulated at 6 h post treatment, with fold changes ranging from 2.35 to 12.52 over controls. Three genes (*SrHDA4*, *SrHDA5*, and *SrHDA10*) showed transient upregulation, with expression returning to near-baseline levels by 12 h. *SrHDA6* exhibited sustained induction, with expression continuing to rise throughout the 12-h period. *SrHDA3* and *SrHDA9* maintained elevated expression at 12 h (3.64- to 3.67-fold over controls), whereas *SrHDA2* declined markedly (to approximately 2.2-fold over controls). These divergent temporal patterns indicate differential MeJA responsiveness among SrHDAC family members.

### 2.8. The Responses of SrHDAC Genes to PEG-Induced Osmotic Stress

**Figure 7:**
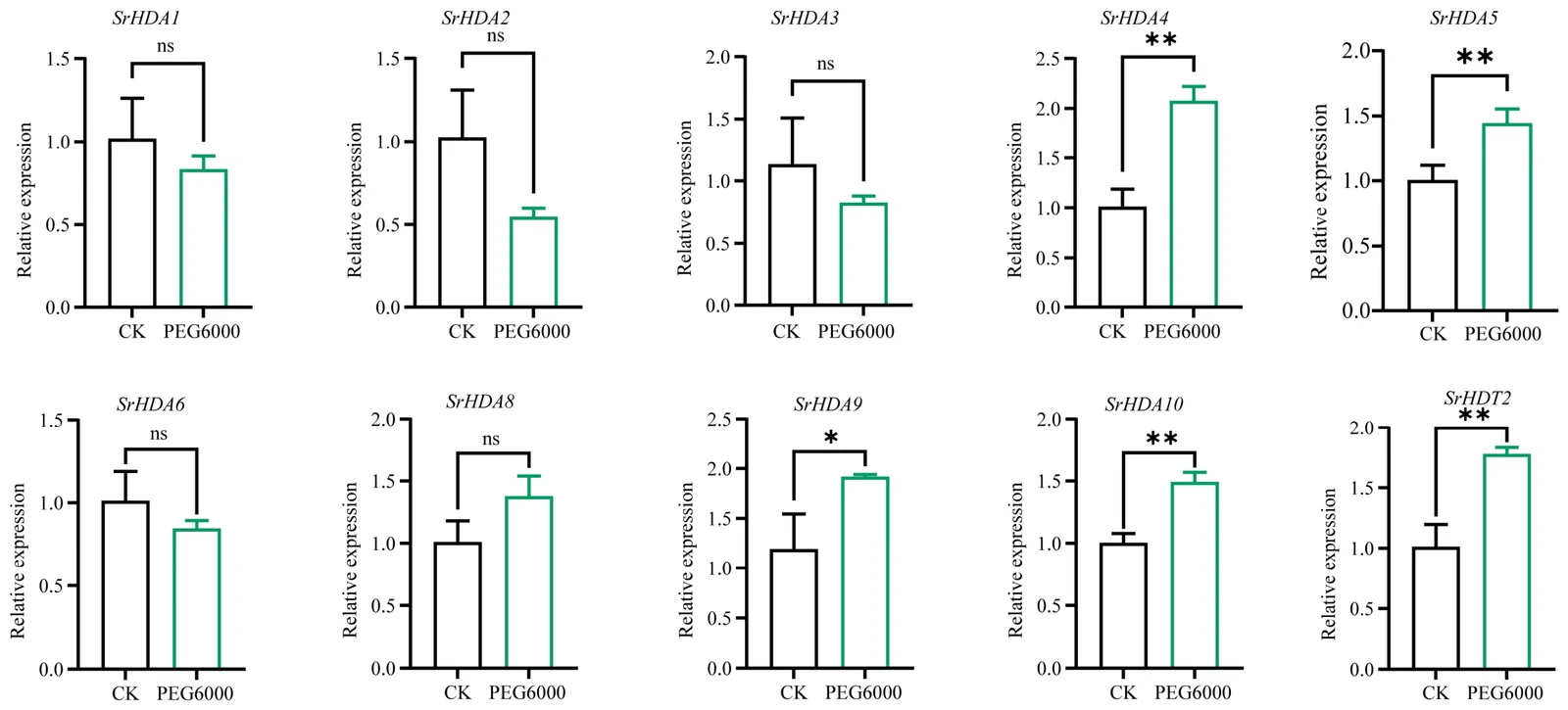
Expression profiles of *SrHDAC* genes under PEG-induced osmotic stress. Relative expression levels of ten *SrHDAC* genes in *S. rebaudiana* seedlings subjected to 20% PEG6000-induced osmotic stress (as a model of water deficit) for 72 h. Expression values were normalized to the untreated control (CK) with *SrActin* as the internal reference gene. Data represent means ± SD (*n* = 3). Statistical significance was determined by Student’s *t*-test; asterisks indicate significant differences (* *p* < 0.05, ** *p* < 0.01) compared to the control, while “ns” denotes non-significant differences.

Expression profiles of ten *SrHDAC* genes following 72 h of 20% PEG6000 treatment, used here as a model of water deficit, revealed differential responses (Figure 7). Five genes (*SrHDA4*, *SrHDA5*, *SrHDA9*, *SrHDA10*, and *SrHDT2*) were significantly upregulated (1.44- to 2.07-fold over controls), whereas three genes (*SrHDA1*, *SrHDA2*, and *SrHDA3*) showed no significant change (Figure 7). *SrHDA6* and *SrHDA8* also exhibited no significant change. These patterns suggest differential participation of *SrHDAC* genes in osmotic stress responses.

### 2.9. Subcellular Localization of SrHDA3.1 and SrHDT1

**Figure 8:**
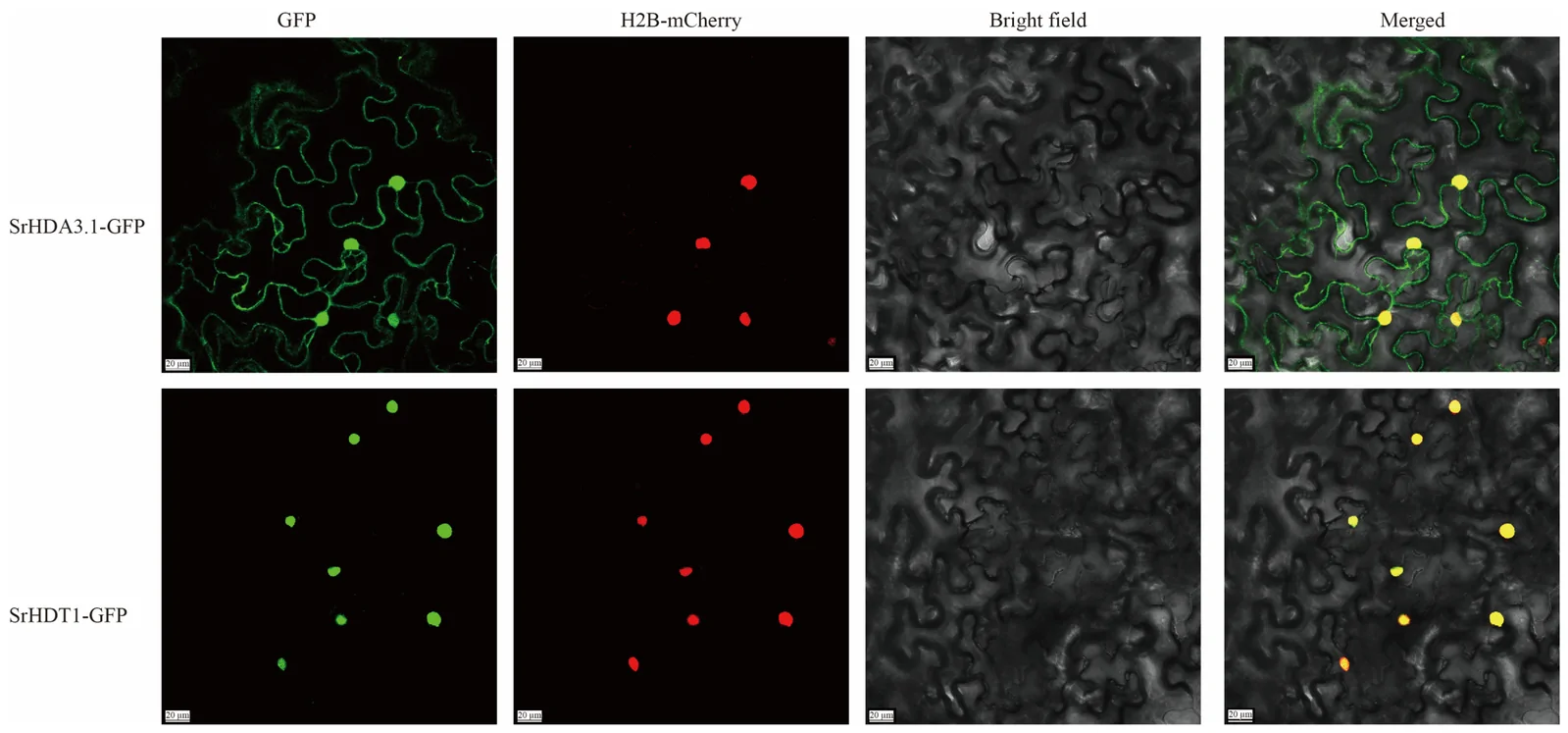
Subcellular localization of SrHDT1 and SrHDA3.1 proteins in *Nicotiana benthamiana* epidermal cells. Confocal microscopy images showing GFP fluorescence (green), nuclear marker H2B-mCherry (red), bright-field, and merged channels for SrHDA3.1-GFP (upper panel) and SrHDT1-GFP (lower panel) fusion proteins. Both proteins exhibit predominant nuclear localization, with SrHDA3.1-GFP showing additional cytoplasmic signals. Yellow signals in the merged images indicate the co-localization of GFP and H2B-mCherry fluorescence. Scale bar = 20 μm.

Transient expression of proteins in *Nicotiana benthamiana* epidermal cells revealed predominant nuclear localization for both SrHDA3.1-GFP and SrHDT1-GFP (Figure 8). Fluorescence signals were concentrated in a subnuclear region consistent with the nucleolus; however, because a nucleolar marker was not included, this interpretation requires confirmation. SrHDA3.1-GFP additionally displayed diffuse cytoplasmic signals, suggesting potential dual localization or nucleocytoplasmic shuttling. These patterns indicate that SrHDA3.1 and SrHDT1 may be associated with nucleolar functions.

## 3. Discussion

### 3.1. Genome-Wide Identification and Classification of SrHDAC Gene Family

We identified 22 *HDAC* transcripts derived from 18 gene loci in the *S. rebaudiana* genome, a repertoire comparable to that of *Arabidopsis thaliana* (18) and rice (18) but smaller than that of apple (26). The sequences were classified into three subfamilies (RPD3/HDA1: 13 sequences from 10 loci; SIR2: 5 sequences from 4 loci; HD2: 4 sequences from 4 loci), consistent with the established plant HDAC classification (Figure 1). This tripartite structure is conserved across angiosperms, suggesting that the three subfamilies originated prior to the divergence of monocots and dicots [2,7,24]. The predominance of the RPD3/HDA1 subfamily (59.1% of identified sequences) mirrors its expansion in other species and likely reflects the fundamental importance of RPD3/HDA1-mediated transcriptional regulation in plant development and stress responses [25,26].

### 3.2. Evolutionary Expansion and Gene Duplication of SrHDACs

Gene duplication is a primary mechanism driving the expansion and functional diversification of gene families. We identified five gene duplication events in the *SrHDAC* family: four tandem duplications (*SrHDT3/SrHDT4*, *SrHDA4/SrHDA5*, *SrHDA6/SrHDA7*, and *SrSRT3/SrSRT4*) and one segmental duplication (*SrHDT1/SrHDT2*) (Figure 3B). All duplicated pairs exhibited Ka/Ks ratios below 1, indicating that they have been subject to purifying selection, which is typical for genes encoding core cellular machinery (Table S2). The prevalence of tandem duplications suggests that local gene duplication has been a major contributor to SrHDAC family expansion. This pattern is consistent with observations in other plants, tandemly duplicated genes are frequently associated with stress responses and secondary metabolism [27,28]. The clustering of *SrHDAC* genes on Chr01 and Chr02 (68.2% of the family) further supports a role for local duplication in shaping the genomic architecture of this family [29,30]. Regarding paralog divergence, the tandem pair *SrHDA4/SrHDA5* showed largely conserved expression patterns, including leaf-preferential expression (Figure 5), transient MeJA induction (Figure 6), and significant upregulation under PEG-induced osmotic stress (Figure 7), consistent with functional redundancy maintained under purifying selection; whether these paralogs have diverged in target-gene specificity or enzymatic activity remains to be tested.

### 3.3. Structural and Functional Diversification of SrHDAC Proteins

The physicochemical diversity of SrHDAC proteins is evident in their broad ranges of molecular weight, isoelectric point (pI), and predicted subcellular localization (Table S1). The variation in pI values (4.55 to 9.14) suggests that different family members may interact with chromatin and associated protein complexes under distinct cellular pH conditions, potentially enabling context-dependent regulatory activities. The predominantly hydrophilic character of SrHDAC proteins (20 of 22 with negative GRAVY values) is consistent with their predicted function as nuclear enzymes operating in an aqueous intracellular environment. Differences in molecular weight may reflect the presence of additional regulatory domains in larger members (e.g., *SrHDA6* and *SrHDA7*) and a more compact catalytic core in smaller members, although domain-level evidence for this inference is currently lacking.

The predicted subcellular localization patterns differed markedly among subfamilies. HD2 members were exclusively predicted to localize to the nucleus, consistent with their established role as transcriptional regulators that directly modulate chromatin accessibility [31]. By contrast, RPD3/HDA1 and SIR2 members were predicted to distribute across chloroplasts, cytoplasm, and nucleus, suggesting that these proteins may have acquired functions extending beyond canonical histone deacetylation [32]. These predictions, generated using computational algorithms, provide testable hypotheses that require experimental verification in native stevia tissues.

### 3.4. Regulatory Mechanisms Inferred from Cis-Acting Elements

Promoter analysis revealed a diverse array of cis-acting elements in *SrHDAC* genes, including light-responsive, hormone-responsive, stress-responsive, and development-related motifs (Figure 4, Table S3). The presence of these elements is consistent with the established roles of HDACs in integrating environmental and developmental signals in plants [11,33]. However, it is critical to recognize that computational prediction of cis-acting elements does not demonstrate functional regulation. The correlation between promoter motif content and observed gene expression patterns is imperfect: for example, *SrSRT2*, *SrSRT3*, *SrSRT4*, and *SrHDT2* promoters contain all five hormone-responsive element types, yet these genes were not included in the qRT-PCR validation panel, and their actual responsiveness to MeJA, ABA, or other hormones remains unknown. Similarly, the presence of drought-responsive elements in certain promoters does not necessarily predict PEG-induced expression changes, as demonstrated by the variable responses of the ten examined genes. These observations underscore the importance of integrating promoter analysis with functional assays to establish causal regulatory relationships.

The MeJA and ABA expression data provide partial support for the regulatory potential implied by promoter elements. Nine of the ten examined genes were upregulated by MeJA, consistent with the abundance of MeJA-responsive elements in their promoters. However, the presence of ABA-responsive elements did not uniformly predict responsiveness to osmotic stress, as some genes with ABA motifs showed no significant change under PEG treatment. This discrepancy highlights the complexity of hormone cross-talk and the limitations of inferring function from promoter sequence alone. We note that a formal quantitative correlation between MeJA-responsive element counts and MeJA-induced fold changes was not attempted, because the ten-gene validation panel provides limited statistical power and cis element number alone does not account for positional context or combinatorial regulation.

### 3.5. Tissue-Specific Expression and Developmental Roles

The tissue-specific expression patterns of *SrHDAC* genes suggest functional specialization across vegetative and reproductive tissues. Leaf-preferential expression of *SrHDA1*, *SrHDA2*, *SrHDA3*, *SrHDA4*, *SrHDA5*, and *SrHDA8* may reflect roles in photosynthesis, leaf development, or secondary metabolite biosynthesis. Given that HDACs modulate secondary metabolite accumulation in other species (*MdHDA6* contributes to drought and salt stress responses in apple through chromatin remodeling [7], and *CsHDA6* mediates the biosynthesis of the anti-insect metabolite α-farnesene through interaction with *CsMYC2* in tea [13]), it is plausible that leaf-expressed *SrHDACs* participate in epigenetic regulation of SG biosynthetic genes. This hypothesis is supported by the presence of light- and hormone-responsive elements in the promoters of leaf-preferential SrHDACs, which may enable coordinated regulation of SG production in response to environmental cues [9]. Flower-preferential expression of *SrHDA6*, *SrHDA9*, *SrHDA10*, and *SrHDT2* suggests involvement in reproductive development, consistent with documented roles of HDACs in floral organ identity and fertility in *Arabidopsis* and rice.

### 3.6. Limitations and Future Perspectives

This study provides the first systematic characterization of the HDAC gene family in *S. rebaudiana*, but several limitations should be acknowledged. First, all functional inferences are based on bioinformatic predictions and transcript-level qRT-PCR data; no genetic manipulation, biochemical assays, or target-gene identification was performed in stevia. Therefore, correlative expression patterns should not be interpreted as confirmed biological functions. Second, osmotic stress was simulated using a single 72-h PEG6000 treatment, which represents osmotic stress rather than soil drought, and may miss early transient transcriptional responses or recovery dynamics. Third, subcellular localization was performed in heterologous *N. benthamiana* cells; the observed nuclear and putative nucleolar patterns require confirmation in native stevia protoplasts or stable transgenic lines. Fourth, expression analysis was conducted for only 10 of 22 identified sequences, leaving the stress responsiveness and tissue specificity of the remaining genes unresolved.

Future studies should prioritize functional validation of key *SrHDACs* through reverse genetic approaches such as CRISPR-Cas9 knockout or overexpression in stevia, combined with transcriptomic and chromatin immunoprecipitation assays to identify downstream target genes. Comparative analysis of HDAC families across multiple Asteraceae species would also clarify the evolutionary trajectory of this family within the lineage. Additionally, investigating the potential role of *SrHDACs* in SG biosynthesis—through co-expression analysis with known SG pathway genes and metabolic profiling of HDAC-modulated lines—represents a promising direction with dual implications for stress tolerance and sweetener yield improvement.

## 4. Materials and Methods

### 4.1. Plant Materials and Growth Conditions

*S. rebaudiana* seeds were obtained from the South China Botanical Garden, Chinese Academy of Sciences (Guangzhou, China). Seeds were surface-sterilized and germinated on Murashige and Skoog (MS) medium (Solarbio, Beijing, China) supplemented with 3% sucrose (Sigma-Aldrich, St. Louis, MO, USA) and 0.5% agar (Solarbio, Beijing, China). Seedlings were transplanted into plastic pots (7 cm × 7 cm) containing a substrate mixture of organic matter, vermiculite, and perlite (3:1:1, *v*/*v*/*v*) and cultivated in a growth chamber under the following conditions: 300 μmol·m^−2^·s^−1^ photosynthetic photon flux density, 12-h light/12-h dark photoperiod, 25 °C/22 °C day/night temperatures, and relative humidity of 70% [34].

### 4.2. Stress Treatments and Sampling

At the five-leaf stage (approximately 4 weeks post transplantation), uniform seedlings were subjected to stress treatments. For MeJA treatment, seedlings were sprayed with 100 μM MeJA (Sigma-Aldrich, St. Louis, MO, USA) containing 0.1% (*v*/*v*) Tween-20 (Sigma-Aldrich, St. Louis, MO, USA); controls received 0.1% Tween-20 only. Leaf samples were collected at 0, 6, and 12 h post treatment. For osmotic stress, seedlings were treated with 20% PEG6000 (Sigma-Aldrich, St. Louis, MO, USA) for 72 h as a model of water deficit; controls were maintained under normal conditions. Leaf samples were collected at 0 and 72 h. All samples were immediately frozen in liquid nitrogen and stored at −80 °C. Each treatment included three biological replicates.

### 4.3. Genome-Wide Identification of SrHDAC Genes

The *S. rebaudiana* genome sequence was downloaded from the Genome Warehouse database (https://ngdc.cncb.ac.cn/gwh/, accessed on 10 August 2025). HMM profiles of the HDAC domain (PF00850) and SIR2 domain (PF02146) were retrieved from the Pfam database and used to search the *S. rebaudiana* protein database using HMMER 3.0 (E-value < 1 × 10^−5^). Candidate sequences were verified using SMART and CDD databases to confirm the presence of conserved domains. Because HD2-type proteins lack the PF00850 catalytic domain, *Arabidopsis* HD2 members (*AtHDT1-AtHDT4*) were additionally used as BLASTP queries, and candidate HD2 proteins were validated by the HD2 domain (PF10243) in Pfam. When multiple transcripts were annotated from a single gene locus (e.g., *SrHDA2.1/SrHDA2.2*, SrHDA10.1/SrHDA10.2), all isoforms meeting the domain criteria (E-value < 1 × 10^−5^ and verified by SMART/CDD) were retained and reported as alternative transcripts of the corresponding locus. All 22 identified transcripts were retained for the phylogenetic and physicochemical analyses. The total count of 22 sequences thus includes both unique loci and alternatively spliced isoforms (Table S1). Physicochemical properties (molecular weight, pI, GRAVY) were calculated using ExPASy ProtParam (https://web.expasy.org/protparam/, accessed on 23 August 2026). Subcellular localization was predicted using WoLF PSORT (https://wolfpsort.hgc.jp/).

### 4.4. Phylogenetic Analysis

Multiple sequence alignment of full-length SrHDAC and *Arabidopsis* HDAC proteins was performed using MAFFT v7.526. A maximum likelihood phylogenetic tree was constructed using IQ-TREE 2 with the VT + G4 + I best-fit substitution model (determined by ModelFinder) with 1000 bootstrap replicates. The circular tree was rendered using Python (Matplotlib v3.11) and annotated in Adobe Illustrator v2020 (Adobe Inc., San Jose, CA, USA).

### 4.5. Gene Structure and Conserved Motif Analysis

Exon–intron organization was extracted from the genome annotation file (GFF3) and visualized using TBtools-II [35]. Conserved motifs were identified using MEME Suite v5.5.7 (maximum motifs = 10, minimum width = 6, maximum width = 50) and annotated using Pfam (v36.0).

### 4.6. Chromosomal Localization and Gene Duplication Analysis

Chromosomal positions of *SrHDAC* genes were mapped from the genome annotation file. Gene duplication events were identified using MCScanX v1.0 [36] with default parameters. Tandem duplications were defined as duplicated genes located within 100 kb of each other on the same chromosome; proximal duplications were defined as duplicated genes located on the same chromosome but separated by more than 100 kb; and segmental duplications were identified by collinearity analysis across different chromosomes. Gene pairs with Ks = 0, for which Ka/Ks is undefined, were excluded [37]; all five retained pairs in the present dataset had quantifiable Ks values (Table S2). Synteny relationships were visualized using TBtools-II.

### 4.7. Cis-Acting Regulatory Element Analysis

The 2000-bp upstream sequences of the transcription start site of each *SrHDAC* genes were extracted using TBtools-II. Cis-acting regulatory elements were predicted using PlantCARE (http://bioinformatics.psb.ugent.be/webtools/plantcare/html/, accessed on 23 August 2026) and categorized into light-responsive, hormone-responsive, stress-responsive, and growth/development-related elements.

### 4.8. RNA Extraction and Quantitative Real-Time PCR (qRT-PCR)

Total RNA was extracted from leaf samples using the RNAprep Pure Plant Kit (Tiangen Biotech, Beijing, China). RNA quality and concentration were assessed using a NanoDrop spectrophotometer (Thermo Fisher Scientific, Wilmington, DE, USA) and agarose gel electrophoresis. First-strand cDNA was synthesized using the PrimeScript RT Reagent Kit (Takara, Dalian, China). qRT-PCR was performed using SYBR Green Premix Ex Taq II (Takara, Dalian, China) on a QuantStudio 6 Flex Real-Time PCR System (Applied Biosystems, Foster City, CA, USA) in a 10 μL reaction containing 5 μL SYBR Green Premix, 0.4 μL each primer (10 μM), 1 μL cDNA, and 3.2 μL RNase-free water. Cycling conditions: 95 °C for 30 s, followed by 40 cycles of 95 °C for 5 s and 60 °C for 30 s. Melting curve analysis was performed to verify primer specificity. *SrActin* was used as the internal reference. Relative expression was calculated using the 2^−ΔΔCT^ method. Ten *SrHDAC* genes were selected for qRT-PCR based on representative membership of the RPD3/HDA1 and HD2 subfamilies, differential promoter cis element composition, and preliminary evidence of tissue-specific or stress-responsive expression. Primer sequences are provided in Table S4.

### 4.9. Subcellular Localization Assay

Coding sequences of representative *SrHDAC* genes (*SrHDA3.1* and *SrHDT1*) without stop codons were amplified and cloned into the pCAMBIA2300-GFP vector to generate C-terminal GFP fusion constructs. Recombinant plasmids were transformed into *Agrobacterium tumefaciens* strain GV3101. *Nicotiana benthamiana* leaves were infiltrated with bacterial suspensions (OD600 = 0.8) and incubated in darkness for 24 h, followed by 48 h of light exposure. GFP fluorescence was observed using a confocal laser scanning microscope (LEICA SP8 STED 3X; Leica Microsystems, Wetzlar, Germany). H2B-mCherry was used as a nuclear marker.

### 4.10. Statistical Analysis

Data are presented as mean ± SD from three biological replicates. For tissue-specific expression (Figure 5), and MeJA time-course experiments (Figure 6), one-way ANOVA followed by LSD post-hoc test was used for multiple comparisons. For the PEG experiment (Figure 7), in which a single treatment group was compared with the control, Student’s *t*-test was used. Significance was defined as * *p* < 0.05, ** *p* < 0.01, and ns (non-significant). Analyses were performed using SPSS v25.0 and GraphPad Prism v10.0.

## 5. Conclusions

This study provides the genome-wide inventory of *HDAC* genes in *S. rebaudiana*, identifying 22 transcripts from 18 loci classified into RPD3/HDA1 (13/10), SIR2 (5/4), and HD2 (4/4) subfamilies. Phylogenetic analysis confirms the conserved tripartite architecture across angiosperms and indicates lineage-specific expansion within the RPD3/HDA1 clade. Five gene duplication events (four tandem and one segmental), all exhibiting Ka/Ks < 1, suggest purifying selection has shaped family diversification. The encoded proteins display considerable physicochemical diversity (MW 18.44–73.72 kDa; pI 4.55–9.14) and are predominantly predicted to localize to the nucleus, chloroplast, or cytoplasm. Subfamily-specific differences in conserved domains, motif composition, and exon number (3–18) point to structural partitioning that may underlie functional divergence. Promoter analysis revealed an enrichment of light-responsive (*n* = 280), MeJA-responsive (*n* = 88), and ABA-responsive (*n* = 72) elements, together with drought-, low-temperature-, and development-associated motifs, implying broad environmental and hormonal sensing capacity. Expression profiling of ten representative genes revealed tissue-specific patterns (six leaf-preferential, four flower-preferential) and indicated that MeJA upregulated nine of ten genes, whereas PEG-induced osmotic stress upregulated five and left five unchanged, suggesting differential participation in stress responses. Subcellular localization assays in *Nicotiana benthamiana* confirmed predominant nuclear localization for both SrHDA3.1 and SrHDT1, with SrHDA3.1 additionally showing cytoplasmic signals. Collectively, these findings establish a foundation for understanding HDAC-mediated epigenetic regulation in *S. rebaudiana* and identify *SrHDA3*, *SrHDA4*, *SrHDA5*, *SrHDA6*, *SrHDA9*, *SrHDA10*, and *SrHDT2* as candidate genes whose stress-responsive expression patterns merit further functional investigation.

## Data Availability

The genome assembly and annotation data analyzed in this study are available in the Genome Warehouse (GWH) of the China National Center for Bioinformation (CNCB), and are publicly accessible at https://ngdc.cncb.ac.cn/gwh (accessed on 10 August 2025). The raw sequencing data have been deposited in the Genome Sequence Archive (GSA) under BioProject accession number PRJCA028196. Any additional data supporting the conclusions of this article will be made available.

## Supplementary Materials

The following supporting information can be downloaded at: https://www.mdpi.com/article/10.3390/ijms27209177/s1.
